# Monitoring event-driven dynamics on Twitter: a case study in Belarus

**DOI:** 10.1007/s43545-022-00330-x

**Published:** 2022-04-08

**Authors:** Natalie M. Rice, Benjamin D. Horne, Catherine A. Luther, Joshua D. Borycz, Suzie L. Allard, Damian J. Ruck, Michael Fitzgerald, Oleg Manaev, Brandon C. Prins, Maureen Taylor, R. Alexander Bentley

**Affiliations:** 1 Center for Information and Communication Studies, University of Tennessee, Knoxville, TN 37996 USA; 2 School of Information Sciences, University of Tennessee, Knoxville, TN 37996 USA; 3 School of Journalism and Electronic Media, University of Tennessee, Knoxville, TN 37996 USA; 4 Stevenson Science and Engineering Library, Vanderbilt University, Nashville, TN 37203 USA; 5 Political Science Department, University Tennessee, Knoxville, TN 37996 USA; 6 School of Communication, University of Technology Sydney, Sydney, NSW Australia; 7 Department of Anthropology, University of Tennessee, Knoxville, TN 37996 USA

**Keywords:** Computational social science, Twitter analysis, Disinformation

## Abstract

Analysts of social media differ in their emphasis on the effects of message content versus social network structure. The balance of these factors may change substantially across time. When a major event occurs, initial independent reactions may give way to more social diffusion of interpretations of the event among different communities, including those committed to disinformation. Here, we explore these dynamics through a case study analysis of the Russian-language Twitter content emerging from Belarus before and after its presidential election of August 9, 2020. From these Russian-language tweets, we extracted a set of topics that characterize the social media data and construct networks to represent the sharing of these topics before and after the election. The case study in Belarus reveals how misinformation can be re-invigorated in discourse through the novelty of a major event. More generally, it suggests how audience networks can shift from influentials dispensing information before an event to a de-centralized sharing of information after it.

**Supplementary Information:**

The online version contains supplementary material available at 10.1007/s43545-022-00330-x.

## Introduction

In the study of social media influence, theoretical perspectives range from behavioral economics to media exposure theory, social “influentials” and their networks, and random contagion dynamics (Aral and Walker 2012; Banerjee et al. 2013; Gleeson et al. 2014; Watts and Dodds 2007; Zajonc 2001). These approaches differ in how much they emphasize the content of messages versus their social networks or media (Acerbi 2020; Bentley et al. 2014; Bond et al. 2012; Carrignon et al. 2019; Lazer et al. 2014). Among those approaches that emphasize social influence, there are contrasting views over the importance of individual, exceptional agents. In one view, influence is heterogeneous and diffuse (Watts and Dodds 2007), such that ideas can emerge from virtually anyone in a network and be shared widely. In this view, there is little value in trying to identify key individuals who lead public information formation.

By contrast, the ‘influentials’ approach assumes that a relatively small fraction of opinion leaders exert influence on a comparatively large fraction of their peers in public opinion formation (Choi 2015; Dubois and Gaffney 2014; Van den Bulte and Joshi 2007). Influence, freely-conferred by followers, reflects the influencer’s own characteristics, such as prestige or status as highly informed, respected, or well-connected (Henrich and Broesch 2011; Watts and Dodds 2007). Since these qualities are judged by the followers, influencers rely on other agents. Represented as a ‘hub-and-spoke’ network structure, the influentials view has been presented, for example, for online terrorist networks, in which influential social media agents have a large number of followers who need not be well-connected to each other (Agarwal et al. 2017; Johnson et al. 2016; Weimann 2015).

The debate is central to the mitigation of online misinformation. On one hand, influential accounts may consistently and repeatedly amplify misinformation (Grinberg et al. 2019; EIPT 2020), such that the removal of one central ‘hub’ dramatically reduces the flow (Albert et al. 2000). On the other hand, misinformation may be diffused by large numbers of smaller-scale agents, such that the removal of smaller groups is needed to weaken the larger ones (Johnson et al. 2019). These models and strategies are not mutually exclusive (Hedström et al. 2000; Rivera et al. 2010); information networks are dynamic and may shift in time between centralized influentials versus diffusive sharing (Stopczynski et al. 2014).

Certain events likely catalyze this shift. Here, we refer to an “event” in the sense that most people would perceive—a national election or protest, for example. This pragmatic definition is subsumed by the more nuanced description of Wagner-Pacifici (2010), in that people perceive a specifiable event as having significant and durable transformational effects. In this sense, an event presents a break in the unremarkable continuity of everyday life (the uneventful world). Events become eventful as they are mediated by news reports, artistic media, and social media feeds (Wagner-Pacifici 2017).

Information about a planned event may initially be coordinated by influential “hubs” in the online network, but once that event has occurred, it may subsequently be shared in a more diffused network. We expect this as a regular pattern, as sharing—including information or gossip—is an evolved human behavior that establishes and maintains social connections (Hrdy 2009; Tomasello et al. 2005; Tomasello 2019; Dunbar 1998; Hess and Hagen 2006). In the U.S. in 2020, for example, older people shared more misinformation (regarding COVID-19) on social media than young people, and yet older people are actually less inclined than young people to believe that misinformation (Lazer et al. 2020).

Events can rejuvenate disinformation campaigns among their audiences, which risk saturation effects if the messages are unchanging. As novelty itself is attractive (O’Dwyer and Kandler 2017), it can be strategic to disseminate enduring themes and narratives in the fresh context of new events. Real-world events can provide new gloss on otherwise consistent broader themes of propaganda (Darczewska 2014; Gerber and Zavisca 2016). During and after the Ukraine crisis of 2014–2015, for example, Soviet-era symbols and narratives were shared on the Russian social media service VKontakte in ways that promoted neo-Soviet myths and nostalgia about World War II (Kozachenko 2019).

**Fig. 1:**
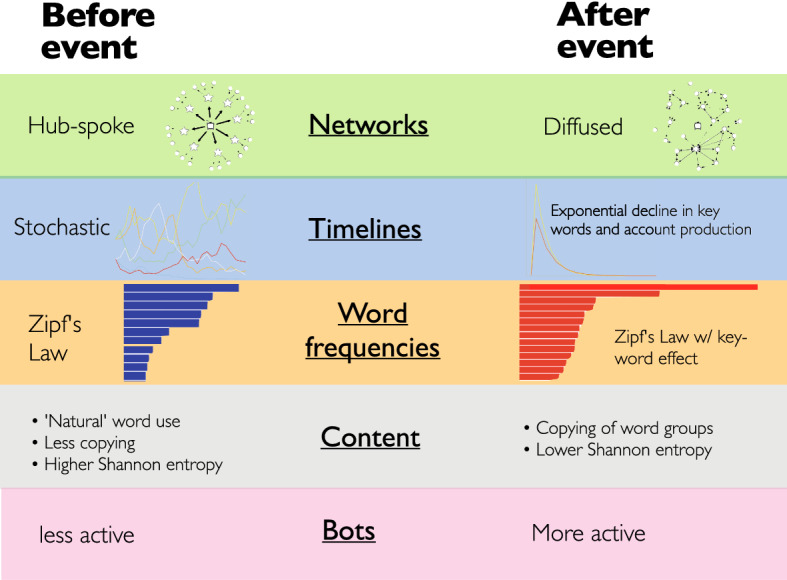
Some expected patterns from Twitter data, in terms of word frequencies and copying networks, before and after a significant event. The network representations are as proposed by Watts and Dodds 2007

In terms of empirical patterns through time, we might expect a two-step process (Fig. 1). First, the lead-up to an event and the immediate response to it, and second, the social-sharing and discussion of the event in terms of long-running themes (Brock et al. 2014; Bentley and O’Brien 2017). Before an event, agents may be focused mainly on centralized information sources. Consequently, the pre-event attention network likely has a “hub-and-spoke” structure (Fig. 1, top left). Immediately after a consequential event is reported by such centralized sources, individuals respond independently to the news. Subsequently, as agents discuss the facts of the event with their peers, the network becomes more clustered and diffuse (Fig. 1, top right). Simultaneously, different clusters of correlated keywords/phrases/hashtags may emerge that contrast with the more centralized “broadcast” network of how the news itself was first disseminated.

Besides the diffusion of network interactions, Fig. 1 shows other patterns in the social media data we might expect to change after a significant event. Before the event, one null expectation is that if we rank words by their frequencies from most common to least common, then those frequencies will decline in a regular relationship known as “Zipf’s Law” (Fig. 1, middle left). After the event, we expect certain words (related to the event) to be emphasized beyond what is expected under Zipf’s Law, so we predict that the top few word frequencies will be unusually high, and then moving down the ranks will fall off abruptly (Fig. 1, middle right). This reflects increased copying of certain words and phrases across the diffused network, so we expect a decrease in a measure of information content known as Shannon entropy (Shannon 1949).

Another pattern to watch for on social media is the increased activity of non-human agents including bots, sockpuppets, news aggregators, or “useful idiots” without malicious intent (Broniatowski et al. 2018; Ferrara et al. 2016; Horne et al. 2019; Kumar et al. 2017; Starbird et al. 2018; Zannettou et al. 2019). We hypothesize that relevant bot activity changes after an event (Fig. 1, bottom). This would depend on the bot-designers strategy, but might generally be expected to be a burst of bot activity, with clusters of nearly identical bots surrounding certain nodes of the network (Johnson et al. 2016; Agarwal et al. 2017). Fake social media accounts, coordinated through common algorithms, often form star-like networks where certain accounts are linked by large numbers of follower accounts who are otherwise disconnected from each other (Johnson et al. 2016; Ruck e al. 2019). These “botnets” can emit synchronized bursts of identical tweets in disconnected networks, “star-like” network structures and other anomalous patterns involving specific hashtags and place names (Agarwal et al. 2017).

We explore these specific five dimensions (retweet networks, timelines, word frequencies, content, and bots) across a major event. Although all of these dimensions have been featured in studies of social media influence, they have not been studied as an ensemble across events. We thought that by examining the five dimensions, we would be able to better discern what might be impacting on the formations of the network structures and the shifts in the digital communication channels. Moreover, since each dimension is quickly and inexpensively quantifiable, we thought these measures may contribute to a toolkit for detecting when social networks are vulnerable to disinformation campaigns. To be robust, a predictive toolkit will require multiple dimensions with strong signals. By studying these dimensions together within a single context, we create a building block for future applied work.

If typical patterns in social media data can be identified, they could be used as null models for judging the magnitude effects of other events. They might also be used to contrast how artificial reactions to news, by bots and algorithms, reveal patterns distinct from human responses to events. The first step is to explore this premise on social media, with a hypothesized pivot point being a substantial event, such as a national election.

## Case study: 2020 Belarusian presidential election

Here, we present some dynamics of pre- and post-event activity using a “natural experiment” among Russian-language Twitter accounts in Belarus, sampled over the 86 days from June 29 to September 23, 2020. We focus on the Belarusian presidential election of August 9, 2020, by monitoring Russian-language Twitter activity before and after this date. The team benefits from members who are native speakers of Russian, whose knowledge was crucial to the qualitative analysis—not just the literal meaning of Russian words in Twitter posts, but perspective in connection to a wider project at University of Tennessee on monitoring and measuring the effectiveness of Russian disinformation in Former Soviet Republics. The ability of the team to consider the results in terms of both content and pattern should reduce researcher bias in the analysis.

We provide brief context to the 2020 Belarusian presidential election (timeline in Supplementary Table S1). As president of independent Belarus, a former Soviet republic, since 1994, Alexander Lukashenko had solidified control over the government and public life, with restrictions on free speech and the press (IREX 2019). Although Belarusian elections between 1996 and 2020 have not been internationally recognized as legitimate (Freedom House 2020), over half of surveyed Belarusians have typically accepted and supported their president (Manaev 2016). In the lead-up to the 2020 presidential election, Belarusian authorities arrested and jailed two challenger candidates, popular blogger Sergei Tikhanovsky and banker Victor Babariko, while a third candidate, Valery Tsepkalo, fled the country. Subsequently, Tikhanovsky’s wife, Svetlana Tikhanovskaya, ran for president in his stead, gaining popularity with support from the campaigns of Babaroko and Tsepkalo. On July 30, over sixty thousand pro-Tikhanovskaya supporters gathered in a Minsk park. On August 9, Lukashenko was officially re-elected with 80.1% of the votes, over 10.1% for Tikhanovskaya. Amid reports of electoral violations (OSCE 2020; Nechepurenko and Higgins 2020), Tikhanovskaya refused to accept the results, fled the country on August 11 and hundreds of thousands of Belarusians protested. Violent clashes with riot police—*омон* in Russian—led to at least one protester being killed and hundreds forcibly detained (Chadwick 2020).

We monitored Russian-language Twitter content and copying networks before and after the 2020 Belarusian election. This project sought to increase methodological replication by gathering the social media sample using a commercially available technology: Salesforce’s *Social Studio* application (www.salesforce.com). This reduced the chance for researcher bias in selecting the sample which could also improve external validity. Using a keyword search in *Social Studio*, Russian-language Twitter data were collected from Belarus during an 86-day period, from June 29 to September 23, 2020 (Table 1). In total, 168,017 Twitter posts were collected. The topics and hashtags selected were based on our monitoring of Russian-language news sources and on content analysis of traditional media sources in Belarus.

**Table 1:** Russian search terms for the elections-related tweets

Search term	translation
Лукашенко	Lukashenko (incumbent president)
Выборы	elections
Цепкало	Tsepkalo (oppositional candidate)
Бабарико	Babariko (oppositional candidate)
9 августа	August 9th (election day)
Тихановский	Tikhanovsky (oppositional candidate)
Тихановская	Tikhanovskaya (oppositional candidate)

Splitting the data into two sets, one before and one after the August election, we analyzed these data in terms of the patterns in Fig. 1. The native Russian-language speaker in our team directed the construction of our own “word bag” of Russian stop words. We processed the Twitter data by removing these stop words. Table 1 shows the keywords used in identifying Russian-language Twitter accounts (see also supplementary Tables S2 and S3). Despite not explicitly stated as part of our model, we also examined the conveyed sentiment of the Twitter data as a component of content by using vector lists of emotion words, or “word bags,” to determine normalized word frequencies, weighted inversely to document frequency. We computed the broad sentiment—positive, neutral, and negative—in Russian by using a neutral network classifier trained on a human-labeled dataset of 18,000 Russian-language news articles and a validation set of 6000 articles (Sakenovich and Zharmagambetov 2016).

Next, we applied unsupervised topic analysis using a Latent Dirichlet Allocation (LDA) topic model (Blei et al. 2003; Grün and Hornik 2018). The LDA topics are based on the co-occurrence of diagnostic two-word phrases across all tweets. Each tweet is considered a mixture of the topics based on frequency (or presence/absence) of the different word pairs. The number of topics is an adjustable parameter of LDA implemented in a Bayesian framework (Cao et al. 2009; Griffiths and Steyvers 2004; Murzintcev 2016). The ideal number of topics, *k*, is that which provides the model with the best topic prediction probability on a held-out sample of words. We ran topic models on all of our data combined, as well as the data collected using the keywords, for Tweets before versus after the August election. We used pyLDAvis (github.com/bmabey/pyLDAvis) to qualitatively explore the identified topics in each data split.

Finally, we visualized the content sharing networks before and after the election, using the text of each tweet and the timestamp of the tweet. Using a method developed previously (Horne et al. 2019), we built a network in a series of steps. First, we built a Term Frequency Inverse Document Frequency (TFIDF) matrix for each 5-day window in the dataset (Horne et al. 2019). Next, we visualized a network, where nodes represent tweets from different accounts and edges connect the tweets whose vector cosine similarity is greater than 0.85 (i.e., nearly the exact same text). The *n* tweets generate *n* completely connected networks of accounts retweeting each message. For each edge in each component, we ordered them by the timestamp by matching each copying tweet to an older tweet with highest cosine similarity. By creating directed edges from the original tweet to the copied tweet, this “pruning” process generated *n* trees (from what had been networks), one for each original source tweet. These trees were then aggregated into a network again, such that the nodes represent Twitter accounts and weighted, directed edges represent the normalized number of tweets copied from one account to another.

## Results

**Fig. 2:**
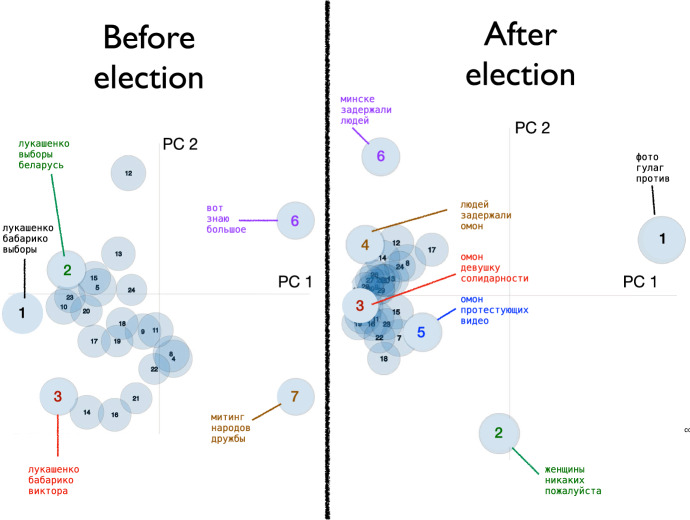
Topic analysis, showing principal component analysis of all topics (PC1 vs PC2), before vs. after the election. Each topic consists of 30 words and their frequencies, but for selected topics, we show the top three words (in terms of mean frequency within the topic. For each term, saliency (Chuang et al. 2012) and relevance (Sievert and Shirley 2014) are visualized using $\lambda = 1$

We examined the observed Twitter patterns while considering the idealized patterns illustrated in Fig. 1. Figure 2 shows the results of the LDA topic analysis for election-related tweets July–August 2020, with each topic characterized by 30 word frequencies. A principal component analysis of these topics represents, in the first two PCs, how related they are to each other.

The change in topics is complex, as each topic represents a unique list of the top 30 most salient words characterizing that topic. Nevertheless, a few broad changes are consistent with the predictions in Fig. 1. First, the majority of topics noticeably cluster together after the election (Fig. 2, right), illustrating the topics across Tweets are less distinctive and less diverse. While we used the same number of topics (parameter *k*) in both topic models for comparison, this heavy clustering post-election suggests fewer topics were discussed after the election than before it (a smaller *k* would provide a better model post-election). One of the words prominent in the tight cluster of topics after the election is “омон” (riot police) which was among the top three words of several topics with the post-election cluster (Fig. 2, right). In contrast, the topics before the election tended to be centered on different candidates, particularly “лукашенко” (Lukashenko), who is the top word in topics 1, 2, and 3 (Fig. 2, left). Overall, again the most notable change after the election is the clustering of topics around the left end of PC 2 (Fig. 2).

**Fig. 3:**
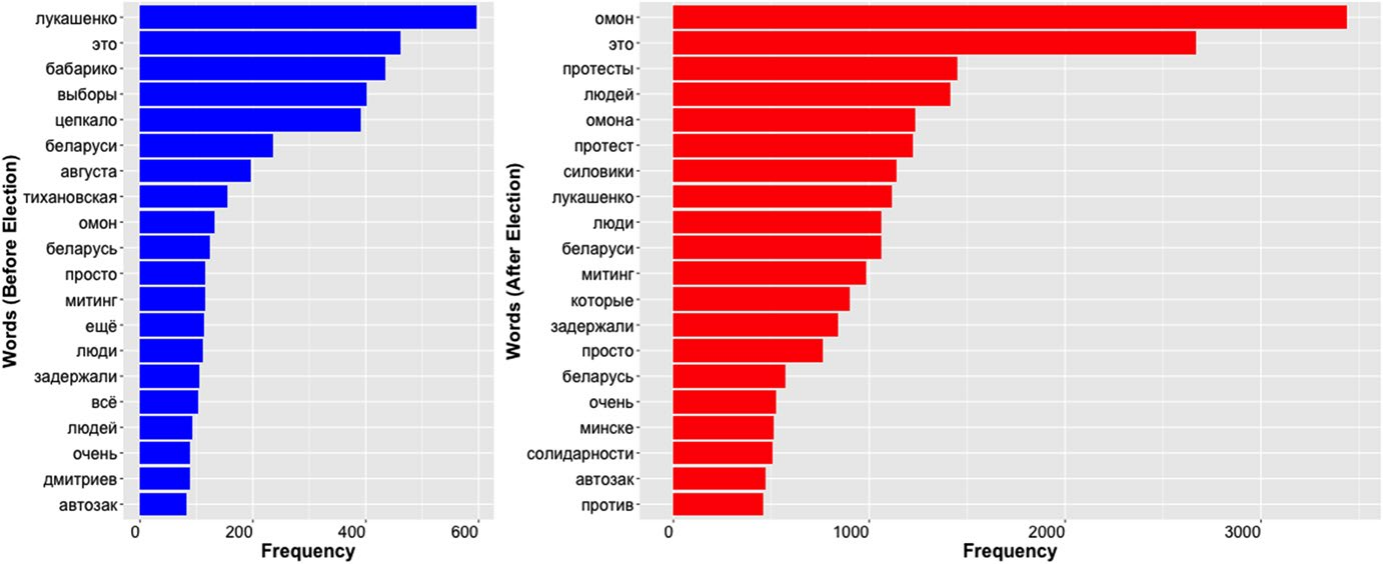
Top 20 words in the Twitter dataset before (left) versus after (right) the election of August 9, 2020

Figure 3 shows the top 20 words in the Tweets before versus after the election. We observe the expected increase in the frequency of the number one and two words after the election (Fig. 1), as well as change in the words occupying these top spots. Before the election, the most common word was the candidate, “бабарико” (Babariko), whereas after the election it was “омон” (riot police) and other words, including “жывебеларусь” (Belarus Live) and “нахуй” (f-word), which spiked on 11 August, just after the election. Notably, on several dates in summer 2020 (July 16, July 29 and August 11), the frequency of “омон” (“riot police”) spiked and then declined exponentially— as indicative of a response to a relevant event (Fig. 1).

**Fig. 4:**
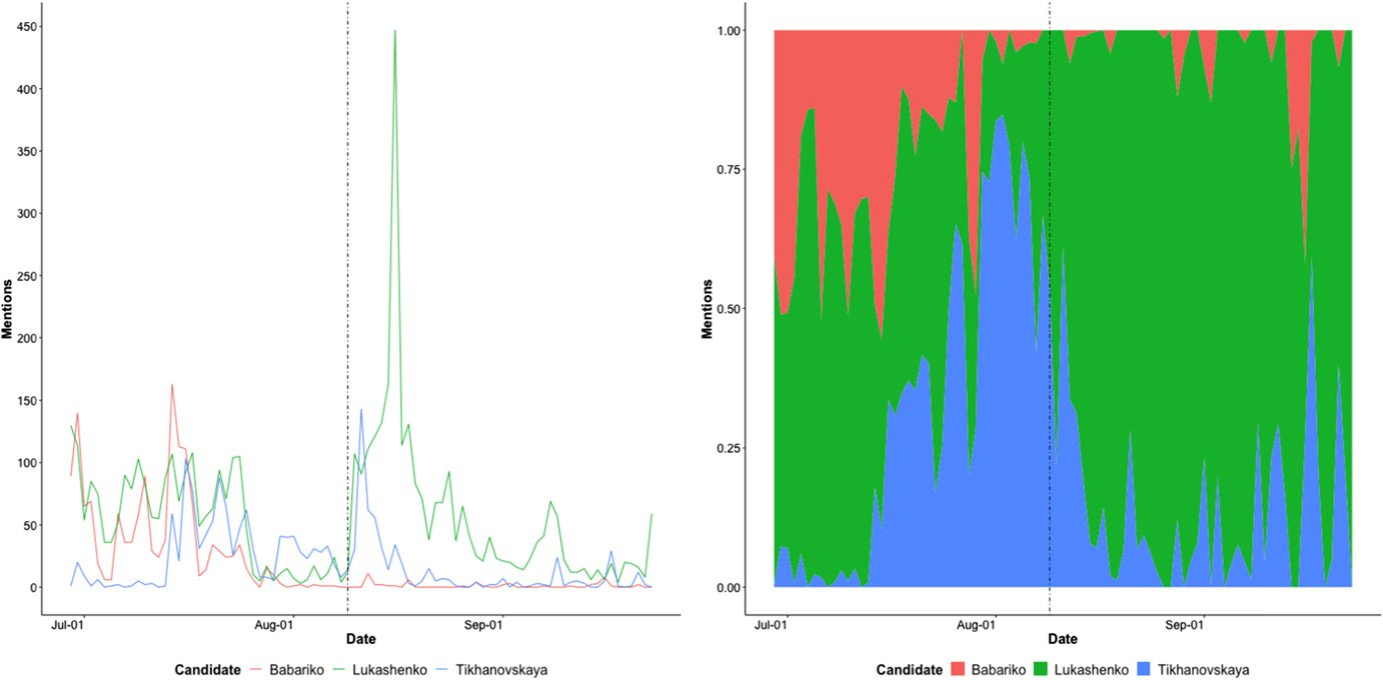
Frequencies of three political candidate names in elections-related tweets. At left are timelines, with dashed line showing the date of the election (August 9, 2020). At right is a fill plot covering the same time period, showing the relative proportions of mentions of each of the three major candidates

Next, we observe timelines. Figure  4 shows timelines of the daily mentions of major political figures in the election-related tweets, namely “лукашенко” (Lukashenko), “Тиханóвская” (Tikhanovskaya) and ‘бабарико” (Babariko). Figure  4, left, shows the spike in mentions of these candidates after the election, followed by an exponential decline, as predicted (Figure  1). When we look at proportional mentions of the three candidates relative to one another (Fig. 4, right), we clearly see the effect of Lukashenko’s election victory. Before the election, mentions of Lukashenko decrease compared to his main opponent, Tikhanovskaya. After winning the election, Lukashenko predominates in terms of proportion of candidate mentions (Fig. 4 right), even as all mentions decline exponentially in raw counts (Fig. 4 left). As we will see in the network analysis, however, there were smaller sites after the election, that maintained support for Tikhanovskaya.

**Fig. 5:**
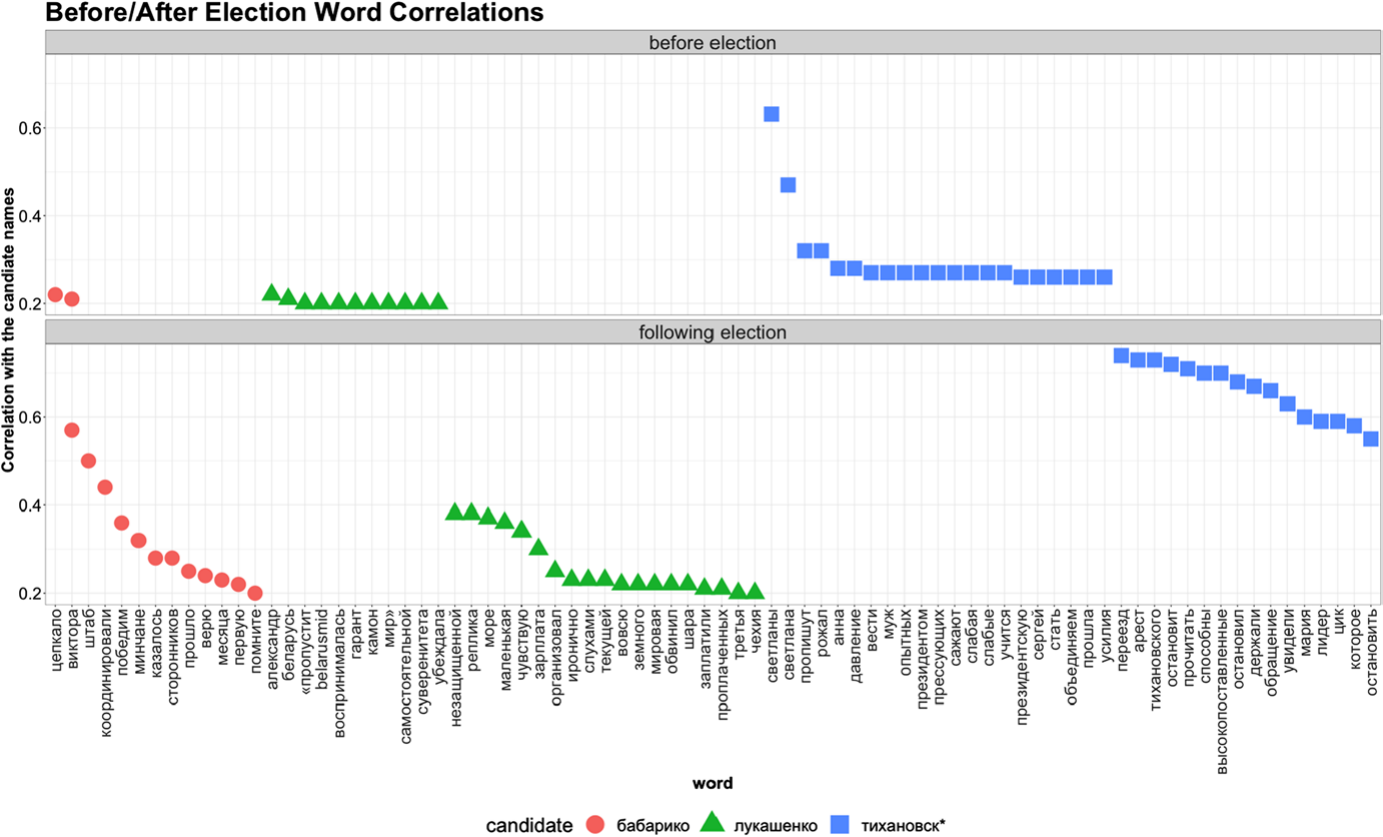
Correlations (Pearson’s $R > 0.20$) between each candidate and the most frequently used words in the same Tweet as the candidate’s name. The top shows before the election, and the bottom shows after the election

Following the examination of candidate mentions, we looked at the relative propensity to copy words through the correlations between the top words associated with each candidate. Our prediction (Fig. 1) is more copying after the event. As Fig. 5 shows, the correlations between each candidate and top words are much stronger after the election, consistent with our prediction. This likely reflects the effect of copying in the discussion of each candidate after the election, with a “signature” of distinct words associated with the candidate, in contrast to before the election when there was more reporting of news associated with each candidate. Notably, there is little overlap of word top lists between candidates either before or after the election (Fig. 5).

**Fig. 6:**
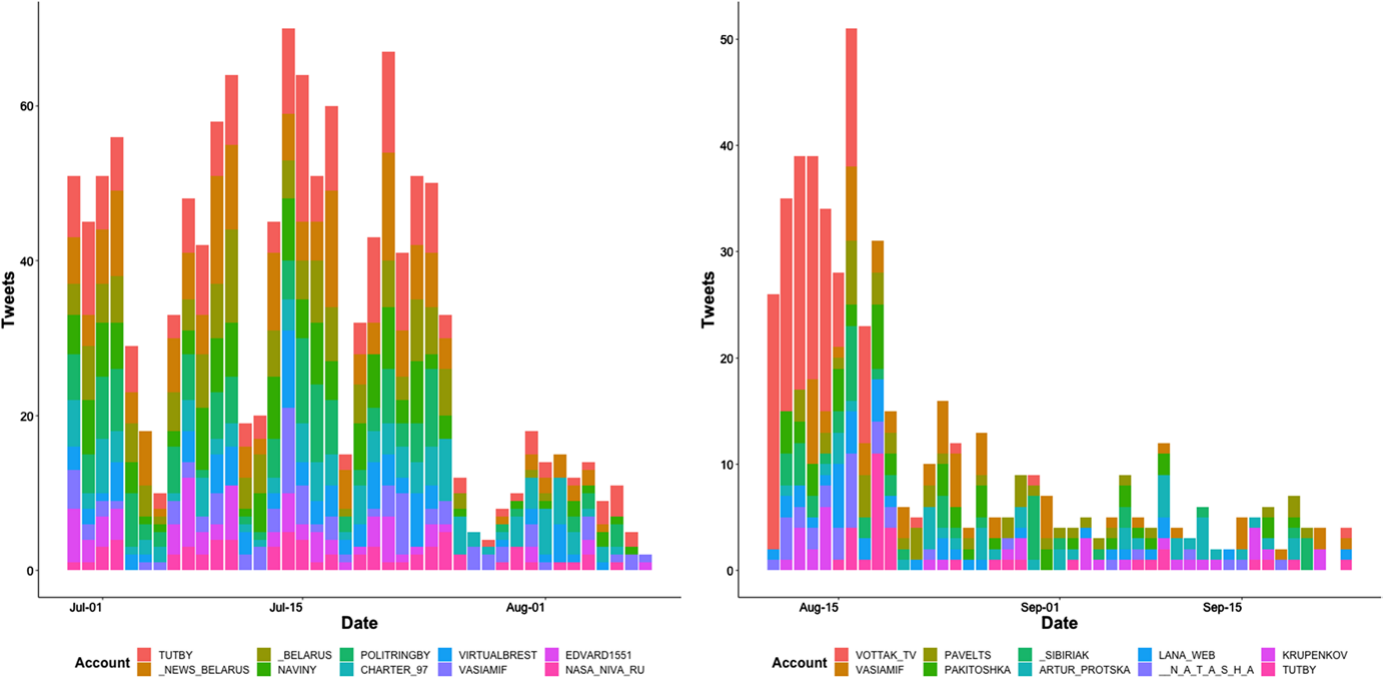
Most productive Twitter accounts that mentioned one of the three election candidates (left) before election and (right) after election

Similar to the pattern in candidate mentions (Fig. 4, left), we see a spike of candidate-related tweets after the election, followed by the expected exponential decline. Figure 6 shows the most productive Twitter accounts, in terms of number of tweets. As anticipated, the most productive accounts before the election were news sites, led by Tut.by, which is among Belarus’s five most popular Russian-language websites, with an estimated 3.5M users in 2020 including over 50% of Internet users in Belarus (IREX 2019). After the election, the most productive accounts included what appear to be bots (Pavelts, Artur Protska, and Patikoshka), given their high tweet rate and relatively few followers, pushing Tut.by down to rank number 10 during the post-election period (Fig. 6 right).

**Fig. 7:**
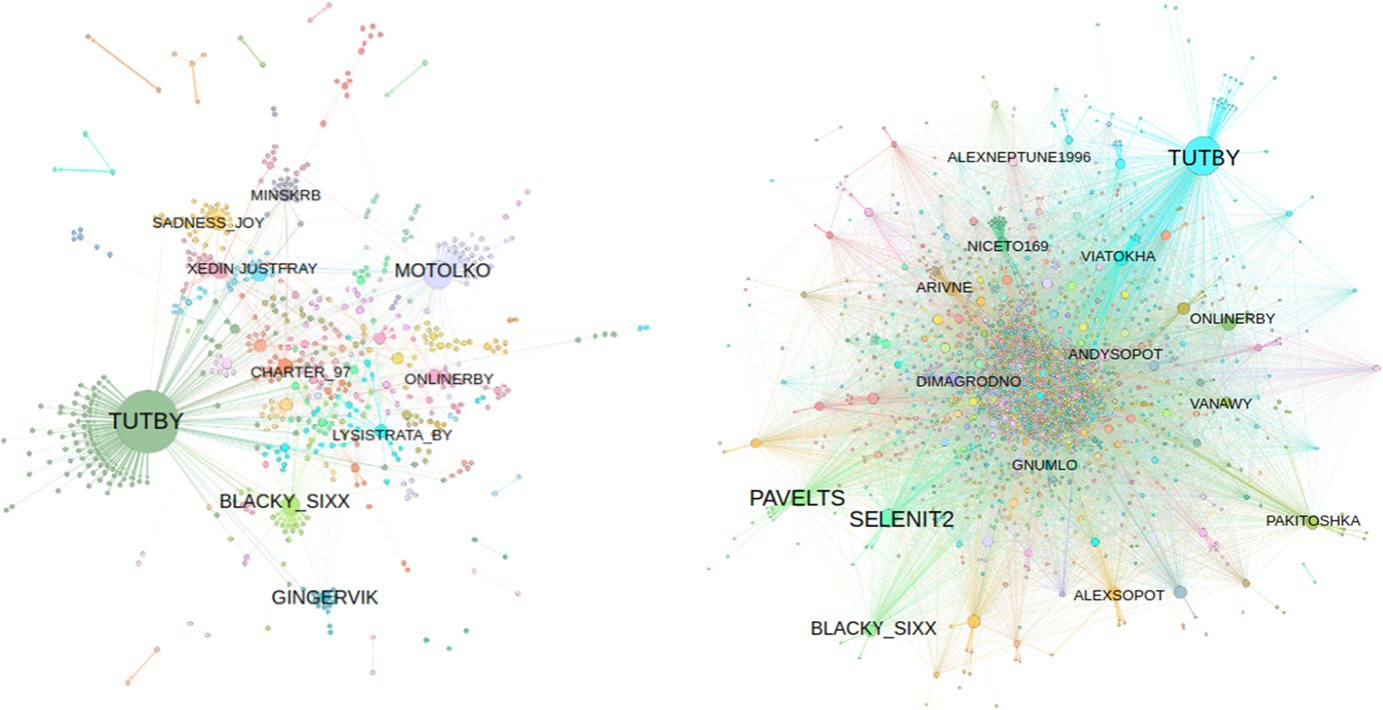
Network of Twitter accounts based on word content, before the election (left) and after the election (right). Colors: community; size: how many copy from the account

**Fig. 8:**
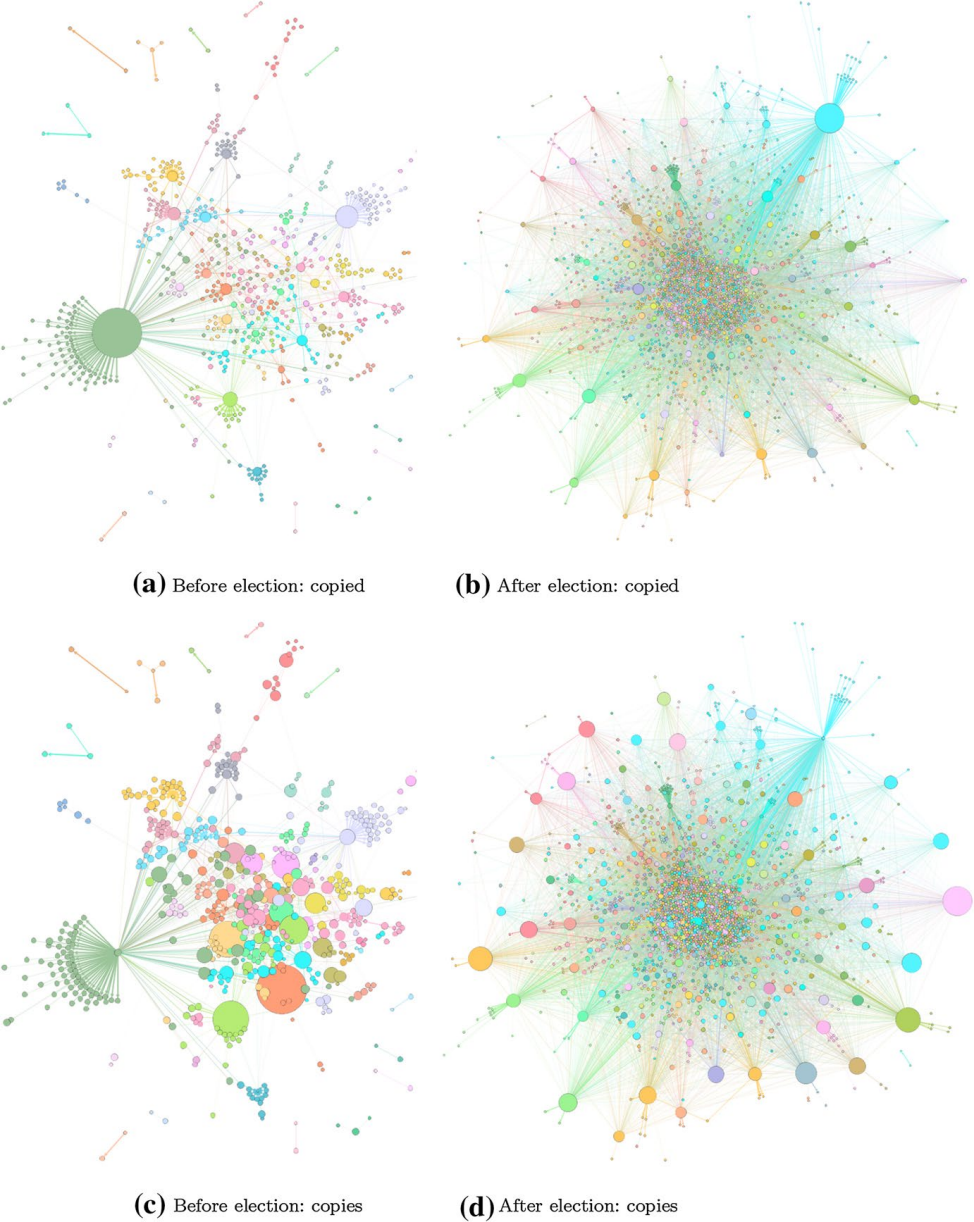
Twitter copying networks before and after the election of 9 Aug 2020. The larger the size of the node, the more that account is copied from (top row) or copies others (bottom row)

**Fig. 9:**
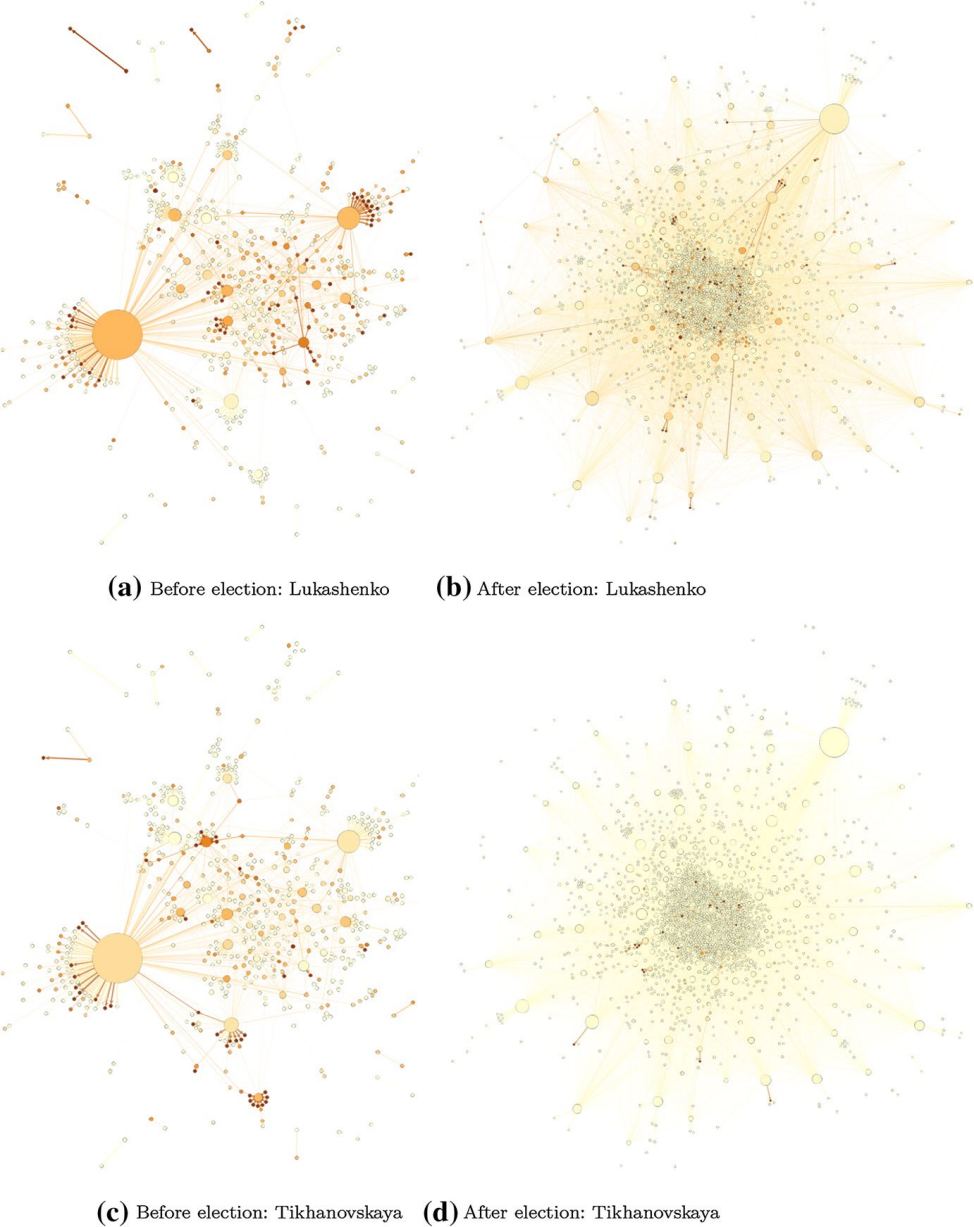
Twitter copying networks before and after the election of 9 Aug 2020. Nodes color indicates proportion of tweets that mention each candidate (darker implies a higher proportion of tweets contained that candidates name)

The changes in tweet-copying networks before versus after the election are visually striking. The election had considerable effect, along the lines predicted in Fig. 1, in how the copying networks change from hub-spoke to diffused. The networks were considerably more centralized before the election, with clear community structures. Afterward, they were de-centralized. Figures 7, 8, and 9 show these networks, which are representations of highly similar text. The networks before the election have well-defined “hubs,” such as Tut.by, which are likely the original (or close to original, due to Twitter API sampling) producers of wide-spreading tweets. The nodes facilitating the spread, however, may not be obvious in these network representations, due to the potential for coordinated cooperation among accounts (Starbird et al. 2018; Horne et al. 2019; Golovchenko et al. 2020; Wilson and Starbird 2020).

Finally, there was little change in average sentiment among the tweets, which averaged neutral in sentiment before and after the election (Supplementary Figs. S4 and S5). This may reflect limitations in our sentiment analysis. First, we may be losing signal by averaging across such a large number of tweets, ultimately compressing any extremely negative or positive sentiment in the data. Second, automatic sentiment detection in the Russian language is understudied. In this work, we used a Russian sentiment classifier that was trained on language from news articles. While this classifier may work on many tweets in our dataset, other tweets may contain language that is much more informal than the language used in news articles. Hence, we may also be misclassifying some extremely positive or negative tweets that use informal or slang language. Further investigation into these limitations is left to future work.

## Discussion

Qualitatively consistent with our model in Fig. 1, we observed a number of changes in the Twitter network before and after the 2020 presidential election in Belarus. The changes included the diffusion of copying networks, the spike and exponential decline of event-related terms, the over-use of most frequent words and the emergence of distinct clusters of words associated with each of the main candidates. The sentiment analysis showed negligible change after the election, which may reflect averaging a large sample. Twitter bot activity appears to have increased after the election. As a general explanation for these changes, we propose that a major event, such as the election, catalyzes a shift from a more “broadcast” pattern, where attention is focused on major news providers, to a more de-centralized process of discussing the news with peers. The initial pattern we have identified is in line with the findings from a study by Lin et al. (2014) that during major planned events capturing shared media and public attention Twitter activity tends to concentrate on elite users such as news providers with large numbers of followers. While retweeting behavior increases during media events, the content being retweeted are produced by the elites (Lin et al. 2014).

In Belarus, 80% of the population has Internet access to independent information, while government exerts control and censorship over the traditional mass media (IREX 2019; Freedom House 2020). In Belarus, TUT.BY has been the country’s most popular independent news source, providing detailed coverage of the election and the protests. As an influential source, TUT.BY helped facilitate the response that would later diffuse to other smaller sources. Surveyed afterward, multiple participants in the 2020 mass protests in Belarus stated they were motivated by news posted by TUT.BY (IREX 2019; Freedom House 2020)^1^. After the election and this immediate response, the role of TUT.BY as an influential hub gave way to a de-centralized social media network. Presumably, as users shared their views about the election and protests, their discussions began to sort into distinct clusters, de-contextualizing the facts of the event in accordance with their respective worldviews (Rutten 2013).

Assuming it is not a sampling issue, bot activity was stronger after, rather than before the August 9th election. If borne out by other studies, this would indicate that disinformation is used to leverage the socially divisive effects of an event, rather than to cause the event itself (cf. Shirky 2011). This strategy can be effective whichever way the event (e.g., an election, a protest) turns out. For example, English-language twitter bots released before the U.S. 2016 presidential election by the Internet Research Agency (IRA), infamous for its bots and disinformation campaigns worldwide (Connell and Vogler 2017; Chivvis 2017), spiked in response to divisive events exacerbating racial and/or political tensions (Ruck e al. 2019). More recently, “anti-woke” content on YouTube has increased by engaging the existing individual preferences of a small but stable percentage of consumers of far-right content, rather than being caused by YouTube recommendations (Homa Hosseinmardi et al. 2021). If it follows that state-sponsored campaigns often aim to aggravate long-standing divisions, rather than try to create new ones, their general activity may be predictable even if the events themselves are unpredictable.

Rather than trying to cause events, state-sponsored disinformation can leverage de-centralized social network communications after an event by fueling existing divisions. The outcome of the August 9th presidential election in Belarus was never in doubt, as national surveys show president Lukashenko’s previous elections since 1994 were all largely accepted by the Belarusian public (Manaev 2016). Given this expectation, state authorities benefit by deploying social media bots to nudge post-discourse toward vilifying and marginalizing opposing voices, including political rivals (in this case Tikhanovsky, Babariko, and Tsepkalo). This “nudge” strategy leverages existing social media users and does not require overt “control of the information environment,” (cf. Reuter and Szakonyi 2015; Tucker et al. 2017). The state can hold more drastic measures in reserve when needed, such as blocking or shutting down independent online news organizations, social media platforms, and search engines for Belarus-based internet users (Stratcom 202).

In this process, the veracity of information may be less important than the social function of sharing it. This may underlie a strategy, observed previously, of Russian bots exploiting politically or socially divisive events (Ruck e al. 2019). Often large in number, bots might be designed as widespread, low-profile catalysts of social sharing. Although the Russian government had taken a cautious public stand on the Belarusian election, it may have directed bots to confuse the message of mass protests against Lukashenko. The Kremlin’s Internet Research Agency (IRA), for example, has programmed social media bots for targeted disinformation campaigns abroad (Chivvis 2017; Connell and Vogler 2017; Ruck e al. 2019).

While our theoretical framework (Fig. 1) invoked a single, planned event, such as an election, it could apply to micro-events within a broader change, such as social movement that advances in punctuated bursts (Horne et al. 2016). These granular events themselves may become incorporated into a larger disinformation campaign (Arif et al. 2018). For this reason, there is need for more fine-grained observation. As we observed negligible sentiment change, future studies should explore sentiments at closer scale. A genuine lack of sentiment change would be notable, as anger and fear are commonly known levers of propaganda (Berger and Milkman 2012; Wollebæk et al. 2019). It may not be easy to change baseline sentiment in a long-term campaign. Another need for granularity involves the constantly evolving use of social media platforms. While this study monitored Twitter as readily available data, future studies will need to monitor multiple social media, as most users have multiple platforms. Twitter represents a small fraction of followers and users of TUT.BY, which has (as of early 2021) over a million users or followers on YouTube, and hundreds of thousands more on Instagram, Telegram, Vkontakte, Facebook, and Odnoklassniki (IREX 2019; Stratcom 202).

It will also be fruitful to observe how users move between platforms when marginalized or blocked by authorities (Johnson et al. 2019). To counter discourse from the 2020 protests, the Belarusian government blocked access of Belarusian internet users to independent online news organizations, social media platforms, search engines, and mobile internet (Stratcom 202). In December 2020, TUT.BY was deprived of its media status by Belarussian authorities. In response, hundreds of thousands of Belarusian social media users have moved to less conspicuous social media platforms, including Telegram (Stratcom 202).

Similarly, Arif et al. (2018) examined both the retweet networks and content flowing across those networks to examine IRA operations during Black Lives Matter discourse on Twitter, finding that information operations use fictitious identities to influence and shape social divisions. These inauthentic accounts, presented as authentic, leveraged an ongoing set of current events (various Black Lives Matters protests in the U.S. at the time) to influence public opinion on the events themselves, as well as opinions on related events, like the 2016 U.S. Presidential Election (Starbird 2018).

Future research can qualitatively asses individual messages within the dynamics described here. State-sponsored disinformation campaigns often seek to deepen cultural divides and undermine trust in democratic institutions (DHS 2019). This can include reviving nationalist mythologies to demonize foreigners (Kozachenko 2019). On the topic of Crimea’s incorporation, for example, bloggers on LiveJournal leveraged deep-rooted divisions between patriots and liberals in Russian society partly through inconsistent use of word connotations relating to the same event (Kravchenko and Valiulina 2020).

## Conclusion

Social media, which have been used for a decade around the world to inform, organize, and coordinate mass opposition to authoritarian governmental control (Reuter and Szakonyi 2015; Shirky 2011; Tucker et al. 2017), are increasingly used to misinform public audiences as counter-measures. Social media disinformation in post-communist countries involves producers, facilitators, and counter-acting online communities (Koinova 2009; Lyons 2006). The case study in Belarus indicates how the effects of influential opinion leaders, versus peer-to-peer sharing, are in dynamic balance in spreading disinformation. The novelty of a major event can lead audience networks to shift from influentials dispensing information before an event to a de-centralized sharing of information after it.

By considering the network dynamics of how information spreads (Badawy et al. 2018; Freelon et al. 2020; Vosoughi et al. 2018; Watts and Dodds 2007), we can expand upon current studies of the effects of disinformation campaigns, which tend to focus the content of propagandist messages, narratives, and themes (Paul and Matthews 2016; Lucas and Pomeranzev 2016). Overall, this case study contributes to our knowledge of the value of using null models for judging the magnitude effects for events. During prolonged events that may be ongoing, comparative models can be captured throughout the event to find moments of clear transition in dynamics, much like a phase transition in classic network traffic models (Ohira and Sawatari 1998).

In the future, use of such baselines to facilitate pattern detection could also be used to look for disruption in a network to uncover events that may be occurring but are not covered by the news, such as increased activity among pernicious actors who are trying to cloak their activities. We envisage a toolbox that allows social media activity to be used as an “Early Warning System” to help reveal what is not easily seen. This could furthermore be the start of a toolkit for assessing the reaction to an event as being artificial or human, and ultimately how we might use this to uncover invisible events by seeing the “vapor trail” they leave behind.

## Supplementary Information

Below is the link to the electronic supplementary material.

Supplementary file 1 (pdf 12688 KB)

## Data Availability

The data used in this paper have been deposited on Harvard Dataverse at: https://doi.org/10.7910/DVN/FOJPBT.

## Footnotes

1 On December 3, 2020, Belarusian authorities deprived the TUT.BY web portal of its legal status of mass media organization.
