# Supplementary material for: Monitoring event-driven dynamics on Twitter: a case study in Belarus
Source: SN Soc Sci. 2022 Apr 8;2(4):36. doi: 10.1007/s43545-022-00330-x (PMC8990676; doi:10.1007/s43545-022-00330-x)
Supplement: Supplementary file 1 — (pdf 12688 KB) [file 43545_2022_330_MOESM1_ESM.pdf]

Supplement for: "Event-driven dynamics of social media: A case study in Belarus"

N.M. Rice[1], B.D. Horne[1], C. Luther[1], J. Borycz[2], S.L. Allard[1], D.J. Ruck[3], M. Fitzgerald[1], O. Manaev[1], B.C. Prins[1], M. Taylor[4], and R.A. Bentley[1]

[1]University of Tennessee, Knoxville, TN, 37996
[2]Vanderbilt University, Nashville, TN 37203
[3]Northeastern University, Boston, MA, 02115 USA
[4]University of Technology Sydney, Australia

Table S1. Activities surrounding Belarus election, May - September 2020.

Date (in 2020)	Activities
May 29	Blogger Sergei Tikhanovsky arrested.
June 18	Victor Babariko arrested.
July 14	Svetlana Tikhanovskaya registered as a presidential candidate.
July 16	Campaigns of Babariko and Tsepkalo support Tikhanovskaya
July 25	Lukashenko accuses both Russia and Poland of "interference" in the presidential campaign.
July 29	Belarus announces arrest of 30+ Russian fighters from private military group Wagner.
July 30	Tikhanovskaya's supporters (60,000+) gather in a Minsk park.
August 9	Election of Lukashenko, Tikhanovskaya refuses to accept. Protests, police response, 3,000+ detained.
August 10	European Union does not recognize the election, denounces violence. 1000 more arrests, one death.
August 11	Tikhanovskaya flees Belarus, women marches, 700 protesters detained, one death, police violence.
August 13	Protests, detained protesters released, evidence of torture posted on social media.
August 17	Tens of thousands in the "National March for Freedom", Lukashenko booed by factory workers.
August 23	Over 100,000 protesters in Minsk with 50,000 'human chain' of solidarity in Lithuania.
August 25	200,000 marchers in Minsk, more protesters detained.
August 27	Putin backs Lukashenko, threatens protesters with potential Russian response.
August 30	Tens of thousands protest on Lukashenko's birthday. Thousands detained.
September 6	Mass Sunday protest, 100,000+ participants, 600 detained.
September 13	Mass Sunday protests, Belarus-Russia military training started.
September 20	Mass Sunday protests, water cannons and armored personnel carriers used against protesters.
September 27	Over 100,000 participants ("March of 97%"); 200+ protesters detained.

Table S2. Top 30 words (about 6% of words) characterizing the top 3 topics in the topic analysis, using $\lambda = 0.8$. English translations in parentheses.

Topic 1	Topic 2	Topic 3
омон (riot police)	митинг (rally)	протесты (protests)
что (what)	тихановской (Tikhanovskaya)	один (one)
нахуй (fu*k)	светланы (svetlana)	есть (there is)
девушку (girl)	минске (Minsk)	тихановской (Tikhanovskaya)
одно (one thing)	тихановская (Tihanovskaya)	лидер (leader)
вечера (evenings)	президенты (presidents)	арест (arrest)
хотели (wanted)	кандидата (candidate)	тихановского (Tikhanovsky)
дома (at home)	народов (peoples)	лукашенко (lukashenka)
перемен (change)	дружбы (friendship)	остановит (will stop)
сидеть (sit)	парке (park)	переезд (moving)
перемены (change)	светлана (svetlana)	остановил (stopped)
видео (video)	всех (of all)	только (only)
жестоко (brutally)	силовики (siloviki)	по (by)
избил (beat)	мирный (peaceful)	данным (data)
жутких (creepy)	проходит (passes)	папа (dad)
самых (most)	поддержку (support)	набрал (typed)
вчерашнего (yesterday's)	протест (protest)	экзитпола (exit poll)
криками (shouts)	людям (people)	заявил (stated)
парня (boyfriend)	пропагандистов (propagandists)	не (not)
жывебеларусь (live belarus)	агитационный (agitational)	беларусь (Belarus)
поддерживают (support)	задержали (detained)	проведения (holding)
использует (uses)	беларуси (Belarus)	посмотрите (take a look)
скорые (ambulances)	сайте (website)	сегодня (Today)
троянских (Trojan)	онлайн (online)	митинга (rally)
коней (horses)	завтра (tomorrow)	республики (republics)
надеюсь (hopefully)	пройдет (will pass)	работу (work)
омона (riot police)	для (for)	идти (go)
пушкинская (Pushkinskaya)	штаба (headquarters)	всех (of all)
кто (Who)	кандидат (candidate)	омона (riot police)
открытые (open)	прошел (passed)	продолжается (continues)

Table S3. Important Russian words in the election-related tweets, as identified by Dr. Rice (some words would be important only in the right context).

1-gram	Count	Translation	Context
лукашенко	21338	Lukashenko	Belarus president
за	11840	for	Indicates support
бабарико	7226	Babariko	Oppositional candidate, jailed
все	6871	all/everyone	universal support or disaproval?
цепкало	5305	Tsepkalo	Oppositional candidate, jailed
тихановская	4970	Tihanovskaya	Oppositional candidate
августа	3863	August	election were August 9
честные	1789	fair	In Tihanovskaya's slogan (on elections)
александр	1339	Alexander	First name of President Lukashenko
news.tut.by	1306	news.tut.by	main independent online news media
дмитриев	960	Dmitriev	Presidential candidate
минске	787	Minsk	capital
тихановскую	774	Tihanovskaya	Oppositional candidate
новые	743	new	Calling for new elections?
подписей	646	signatures	Signatures register a presidential candidate
видео	626	video	Includes videos of police brutality
ермошина	543	Yermoshina	Head of electoral commission
1994	421	1994	Year Lukashenko elected
тихановского	334	Tikhanovsky	Former candidate, jailed
победила	322	won	Won, by female
белоруссии	319	Belorussia	Old Russian name for Belarus
тихановский	316	Tikhanovsky	Former candidate, jailed
канопацкая	311	Kanopatskaya	Presidential "potemkin village" candidate
одной	308	one	A female
валерия	304	Valeria	Wife of exiled candidate Tsepkalo
tutby	296	tut.by	popular online independent mass media
онт	290	ONT	State-run TV channel
боевиков	285	rioters/mercenaries	Propagandistic term
tut.by	284	tut.by	Online independent mass media
brest.by	226	brest.by	Online news source
ермошиной	223	Yermoshina	Head of electoral commission
чвк	221	Private military co.	Detainers of Russian PMC members
майдан	207	Maidan	Ukrainian location of 2014 revolution
светлану	202	Svetlana	Svetlana Tihanovskaya
силовики	198	siloviki	Security & military forces
григорьевич	191	Grigorievich	A. Lukashenko's patronim
газпром	186	GazProm	poweful Russian bank, head jailed
белгазпромбанка	174	GazProm	poweful Russian bank, head jailed
колесниковой	169	Kolesnikova	top aide to Tihanovskaya
бчб	156	flag (white & red)	Oppositional symbol

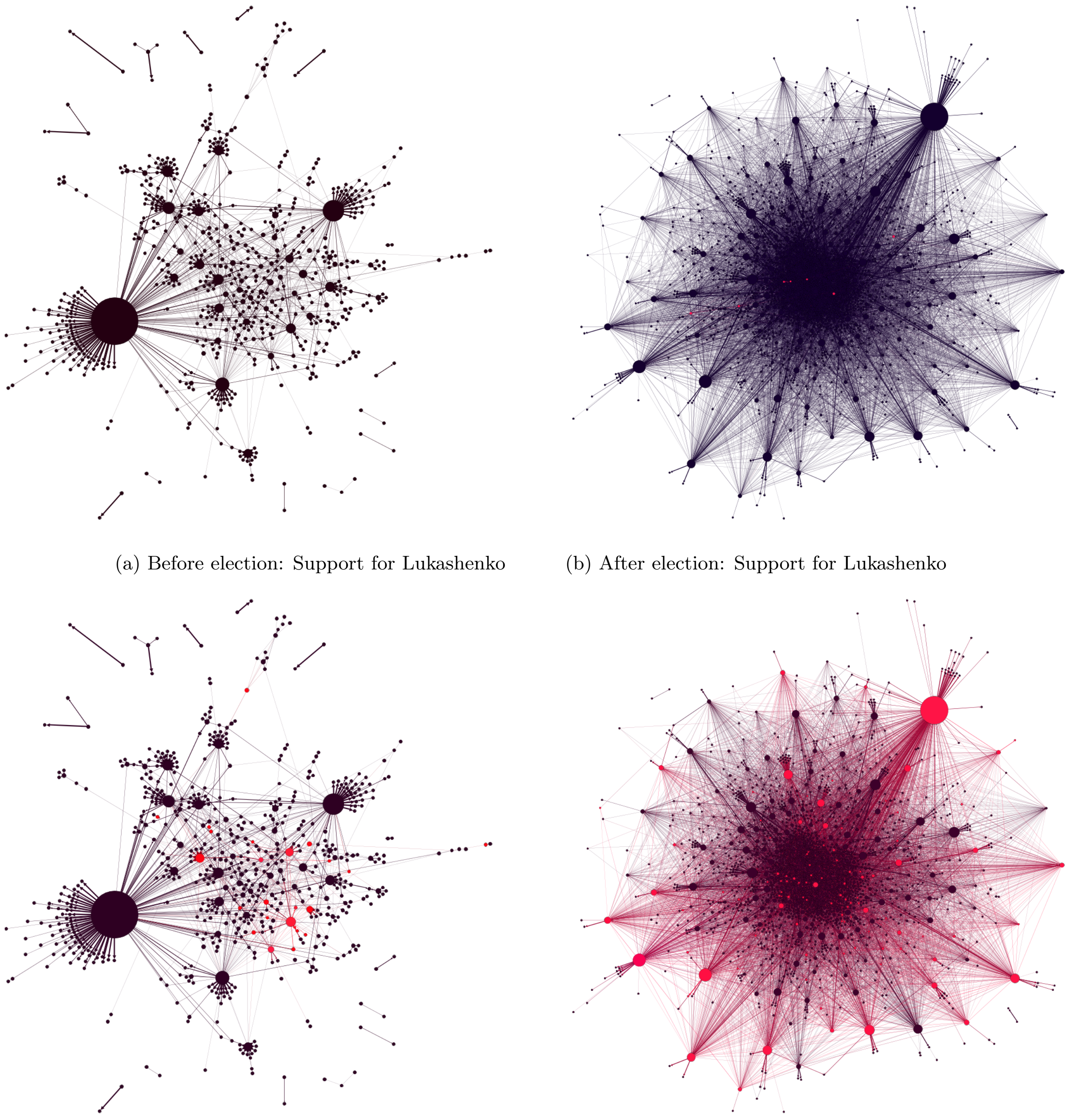

(a) Before election: Support for Lukashenko

(b) After election: Support for Lukashenko

(c) Before election: Support for Tikhanovskaya

(d) After election: Support for Tikhanovskaya

Figure S1. Twitter copying networks before and after the election. Red: account used a support phrase for the candidate at least once; black: account did not use support phrase.

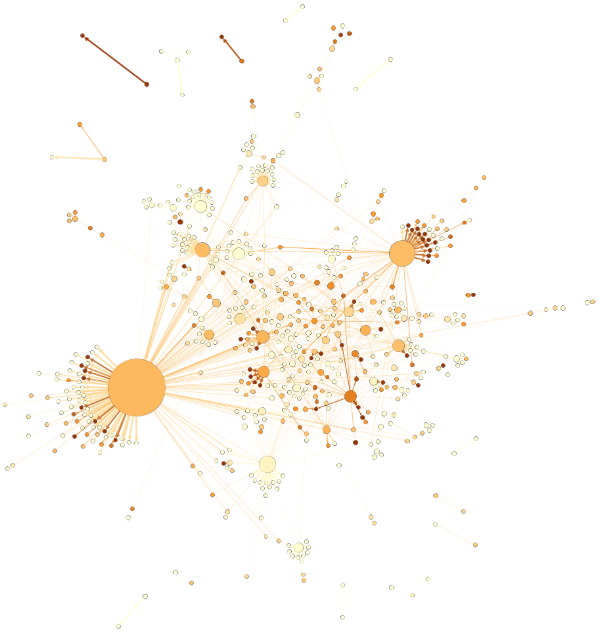

(a) Lukashenko before: proportional

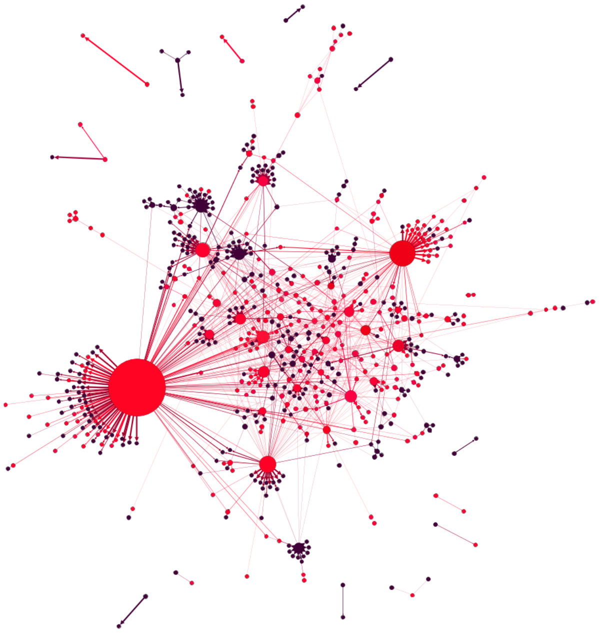

(b) Lukashenko before: binary

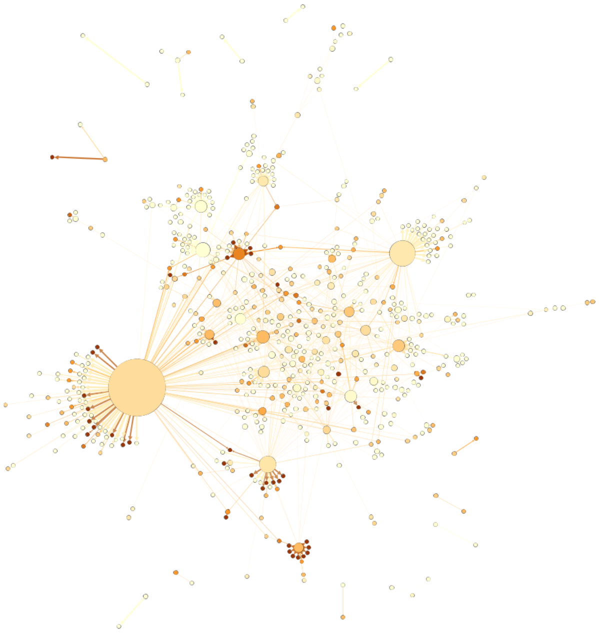

(c) Tikhanovskaya before: proportional

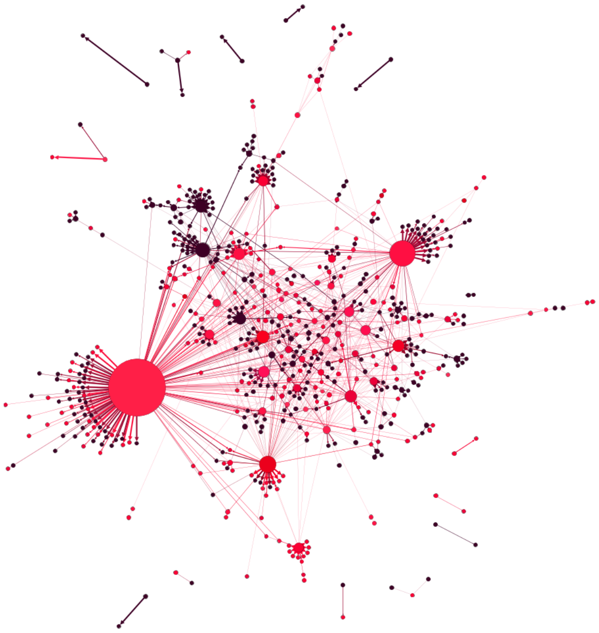

(d) Tikhanovskaya before: binary

Figure S2. Twitter copying networks before the election. Left column: darker implies a higher proportion of tweets contained that candidate's name. Right column: red nodes mentioned candidate, black nodes did not mention.

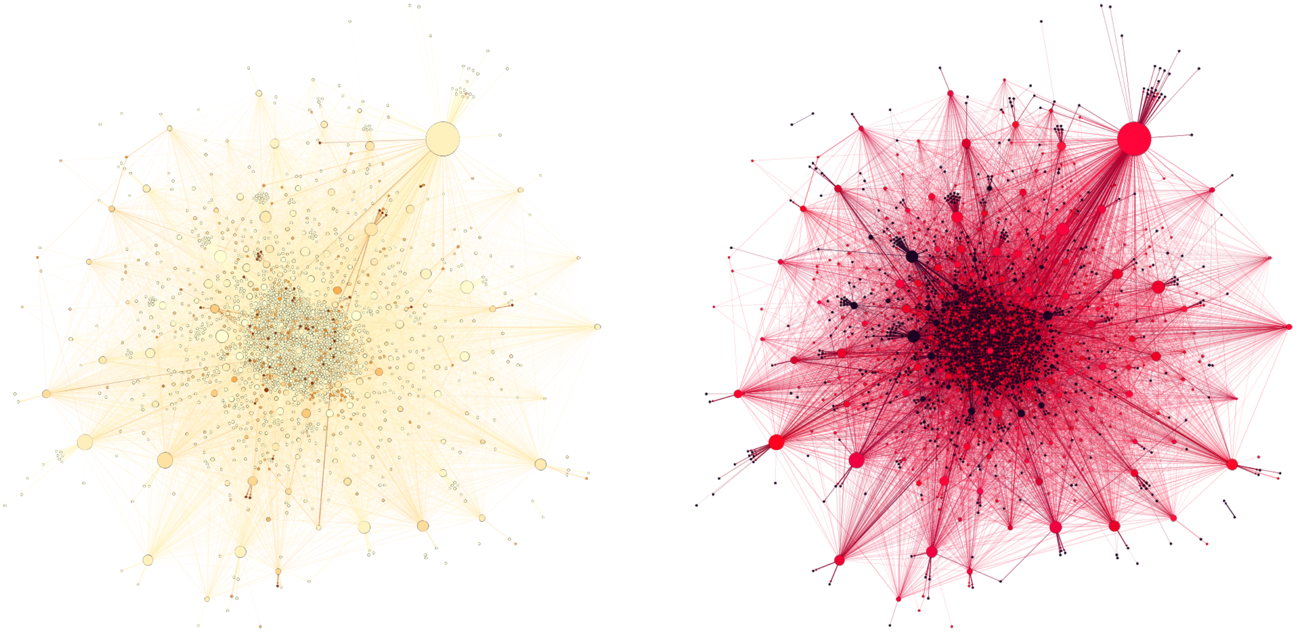

(a) Lukashenko after: proportional

(b) Lukashenko after: binary

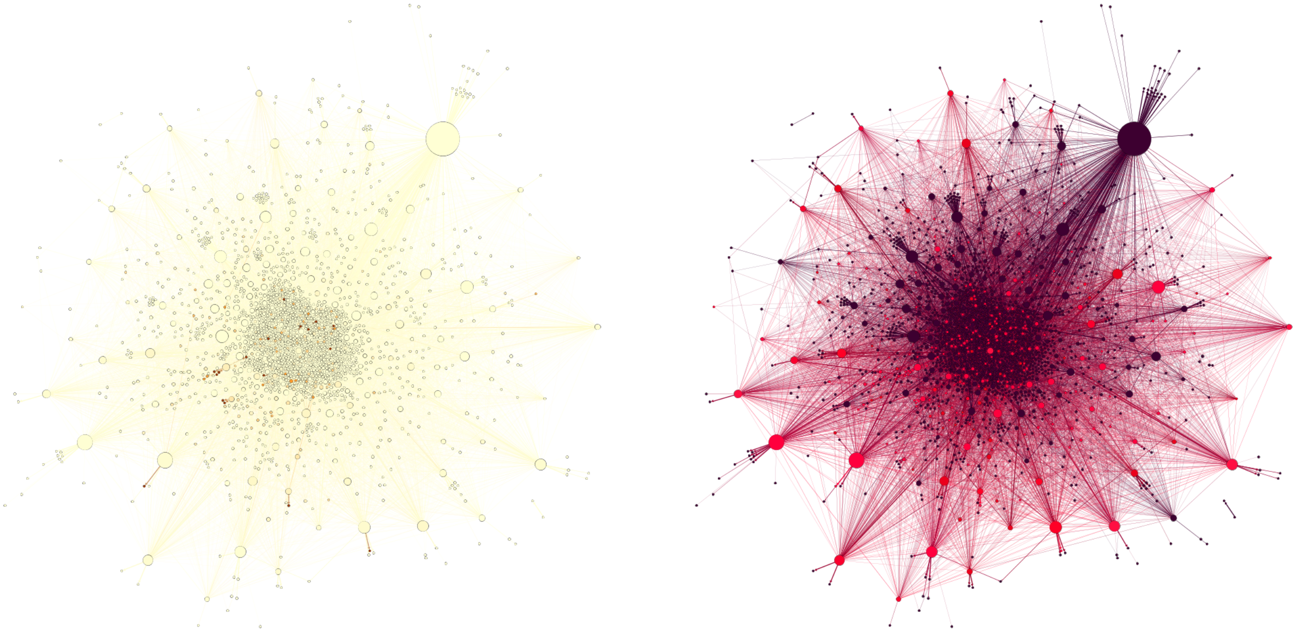

(c) Tikhanovskaya after: proportional

(d) Tikhanovskaya after: binary

Figure S3. Twitter copying networks after the election. Left column: darker implies a higher proportion of tweets contained that candidate's name. Right column: red nodes mentioned candidate, black nodes did not mention.

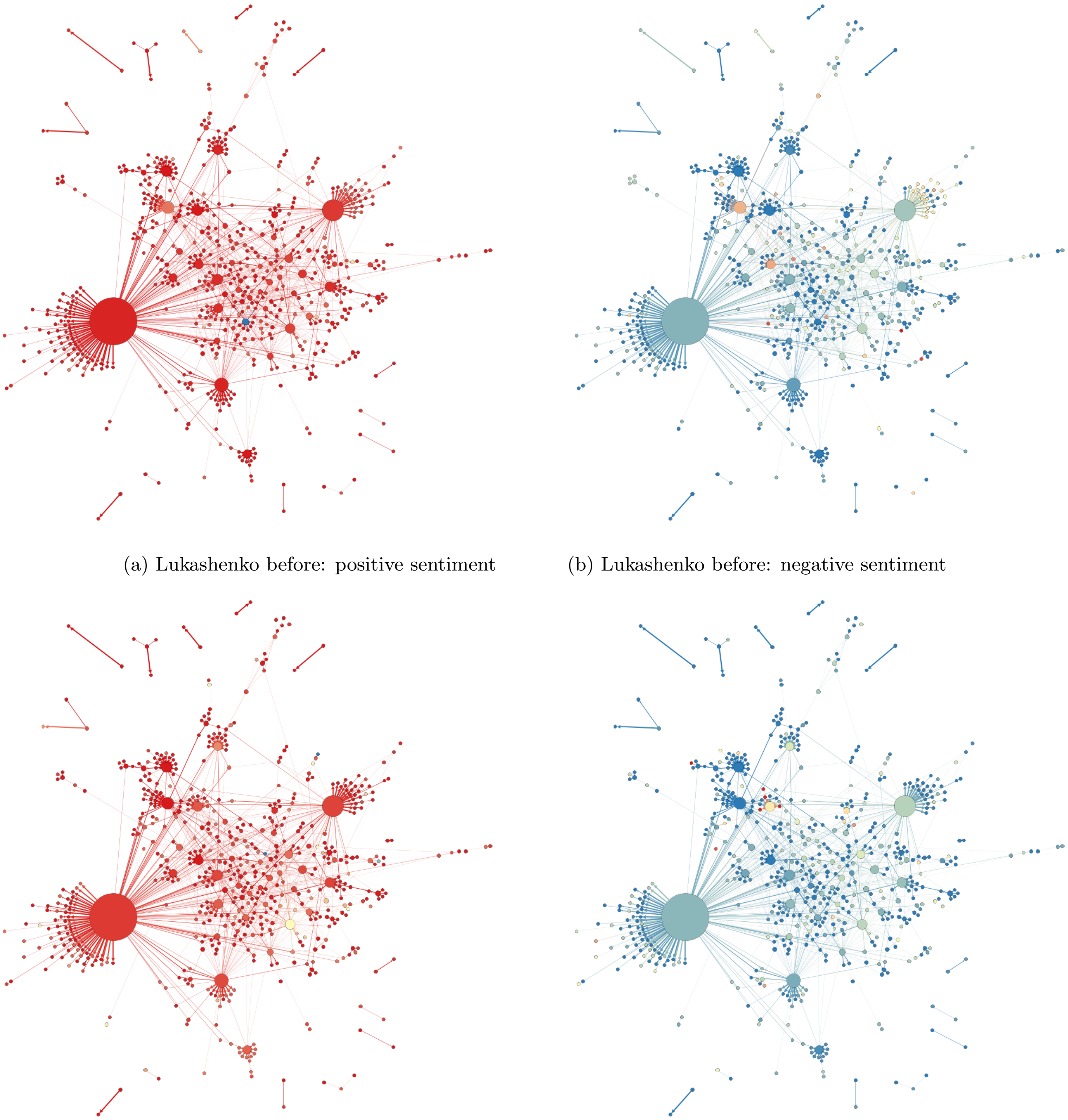

(a) Lukashenko before: positive sentiment

(b) Lukashenko before: negative sentiment

(c) Tikhanovskaya before: positive sentiment

(d) Tikhanovskaya before: negative sentiment

Figure S4. Sentiments in Twitter copying networks before the election. Left column: bluer nodes indicate more positive average sentiment around the candidate; redder nodes are negative or neutral. Right column: red indicates more negative, blue indicates positive or neutral.

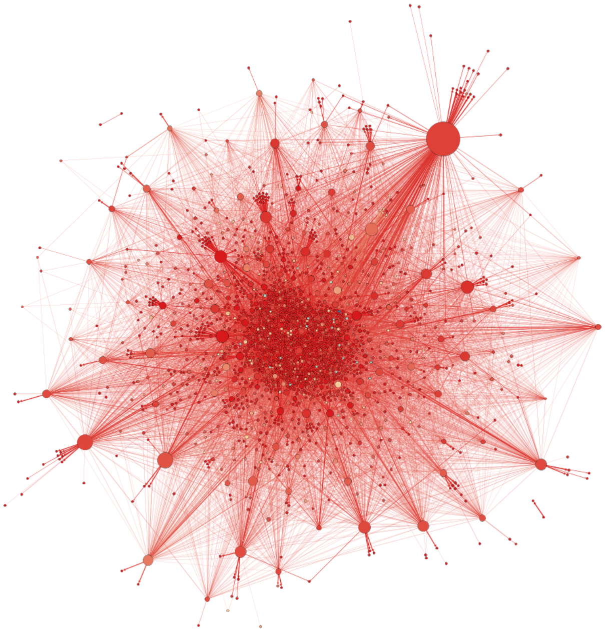

(a) Lukashenko after: positive sentiment

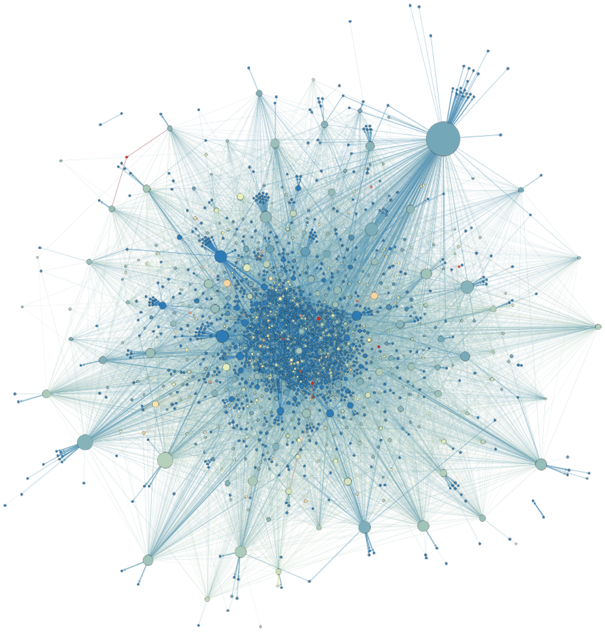

(b) Lukashenko after: negative sentiment

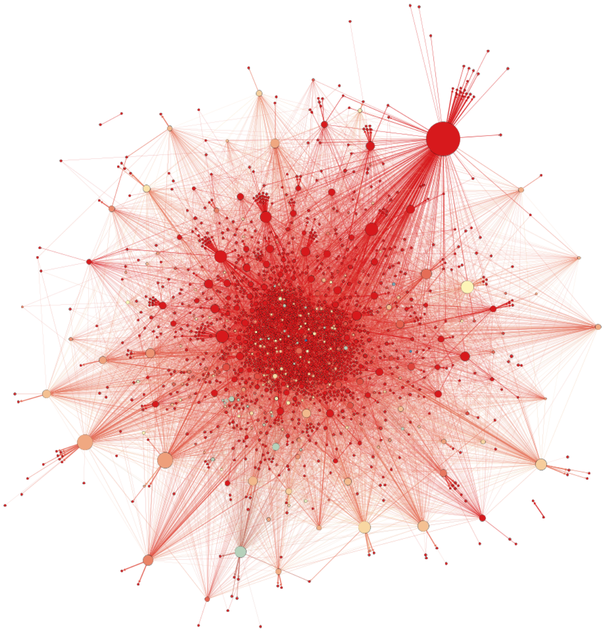

(c) Tikhanovskaya after: positive sentiment

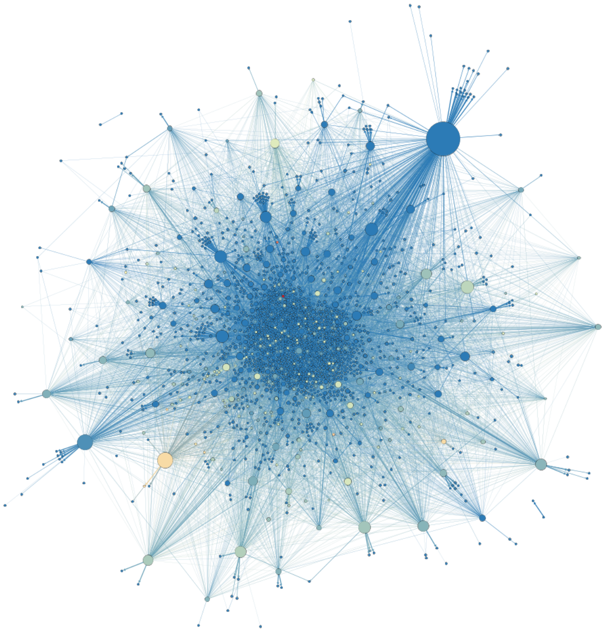

(d) Tikhanovskaya after: negative sentiment

Figure S5. Sentiments in Twitter copying networks after the election. Left column: bluer nodes indicate more positive average sentiment around the candidate; redder nodes are negative or neutral. Right column: red indicates more negative, blue indicates positive or neutral.
